# Melatonin-Based Therapeutics for Neuroprotection in Stroke

**DOI:** 10.3390/ijms14058924

**Published:** 2013-04-25

**Authors:** Kazutaka Shinozuka, Meaghan Staples, Cesar V. Borlongan

**Affiliations:** Center of Excellence for Aging & Brain Repair, Department of Neurosurgery and Brain Repair, University of South Florida College of Medicine, 12901 Bruce B. Downs Blvd., Tampa, FL 33612, USA; E-Mails: kshinozu@health.usf.edu (K.S.); meaghans@mail.usf.edu (M.S.)

**Keywords:** oxidative stress, stroke, neuroprotection, cerebral ischemia, melatonin

## Abstract

The present review paper supports the approach to deliver melatonin and to target melatonin receptors for neuroprotection in stroke. We discuss laboratory evidence demonstrating neuroprotective effects of exogenous melatonin treatment and transplantation of melatonin-secreting cells in stroke. In addition, we describe a novel mechanism of action underlying the therapeutic benefits of stem cell therapy in stroke, implicating the role of melatonin receptors. As we envision the clinical entry of melatonin-based therapeutics, we discuss translational experiments that warrant consideration to reveal an optimal melatonin treatment strategy that is safe and effective for human application.

## 1. Introduction

Melatonin is a hormone produced in the pineal gland, which has long been established as primary modulator of circadian rhythms in mammals [1–4]. Over the last decade, however, melatonin has emerged as a very powerful free radical scavenger and antioxidant [5–8].

Oxidative stress, characterized by increased free radical damage, has been implicated in neurological disorders [9–11], suggesting potential therapeutic benefits of treatment with free radical scavengers (e.g., deprenyl, 7-nitroindazole, iron chelator, vitamin E). Indeed, free radical scavengers and antioxidants protect against cell death [12,13]. Because oxidative stress has been shown in the laboratory to exacerbate stroke-induced pathophysiological and behavioral dysfunctions, the use of free radical scavengers and antioxidants may prove effective in preventing such deficits. To date, many antioxidants have been tested in experimental stroke models and have reached clinical trials (Tables 1 and 2). In comparison with these substances, melatonin has an obvious advantage because it is an endogenous substance. Alternatively, transplantation of melatonin-secreting cells into the ischemic area may allow a novel melatonin-based treatment for stroke, as we will review later.

A number of studies have reported the important role of melatonin on neuroprotection in animal models of stroke. Experimentally induced stroke is exacerbated in pinealectomized rats [39,40]. Melatonin administration after experimental stroke reduces infarction volume [41,42]. Such a protective effect is seen in both gray and white matter [43]. Melatonin also reduces inflammatory response [44], cerebral edema formation [45], and blood-brain barrier permeability [46]. Functionally, melatonin administration improves grip strength and motor coordination, and attenuates hyperactivity and anxiety [47]. Melatonin secretion is known to decrease age dependently [48], suggesting that if melatonin directly affects stroke then aged people should suffer more strongly from insults of stroke. This may also be ameliorated with melatonin pretreatment; studies in animal models of stroke have demonstrated that pretreatment of melatonin exerts anti-inflammatory effects and reduces infarction volume [49–51].

We have demonstrated that chronic exogenous treatment with melatonin protects against experimental stroke [52]. In addition, we have demonstrated transplantation of the pineal gland to experimental stroke rats promotes neuroprotection [53]. Recently, we have further revealed that melatonin receptor type 1A (MT1) is involved in the mechanism of action for neuroprotective effects of stem cells in *in vivo* models of stroke [54]. In this review, we discuss the neuroprotective effects of melatonin with a focus on our studies and other related studies. Thereafter, we also discuss key translational research needed to facilitate clinical trials of melatonin treatment and transplantation of melatonin-secreting cells.

## 2. Glial Cell Protection by Melatonin in Ischemic Brain

The role of glial cells in integrity and degeneration of the central nervous system has shifted from being a mere bystander cells to being actively involved in homeostasis and brain repair after injury. A large body of laboratory evidence has documented that glial cells are critical to neuronal survival [55–58]. In the developing central nervous system, astrocytes have been shown to correctly guide migration and proliferation of neurons, whereas in the adult, astrocytes have been implicated in the maintenance of neuronal homeostasis and synaptic plasticity [55,56]. Astrocytes have been demonstrated *in vitro* to possess receptors [57,58], as well as signaling molecules that can trigger neuronal messages that are key to cell survival [59] or death [60]. Based on this knowledge of the active glial cell role in brain function, examination of cell death that was primarily investigated using pure neuronal cell cultures has now accommodated mixed astrocyte-neuronal cultures that resemble the *in vivo* condition and promote better neuronal survival than pure neuronal cultures [61,62].

Along this line of glial cell’s key participation in neuronal survival and brain function, the identification of trophic factors, such as glial cell-line derived neurotrophic factor (GDNF) [63], has prompted investigations into the possible therapeutic actions of glial cells. Glial cells are the main source of transforming growth factor b (in which GDNF is a subfamily member) and astrocytes have been shown to release many growth factors under normal conditions or in response to brain injury [64,65]. Accordingly, experimental treatment strategies for neurodegenerative disorders such as Parkinson’s disease have exploited the support and trophic factor properties of glial cells [66]. For example, a major outcome assay of successful cell therapy in Parkinson’s disease, is the characterization of surviving donor glial cells remaining proximal to the grafted site [67,68], allowing axons of passage to reach host targets [69]. Moreover, transplantation of astrocytes transfected with the gene responsible for synthesizing and secreting GDNF, or the dopamine precursor l-DOPA, have been demonstrated to provide enhanced amelioration of parkinsonian symptoms [70]. Grafted embryonic dopaminergic neurons combined with an infusion of astrocytic growth factor, or GDNF have similar positive effects. Additional properties of astrocytes include their ability to control water balance, and to reduce glutamate toxicity [71,72]. Astrocytes siphon excess extracellular water and potassium ions, which are then redistributed to their networks or excreted into the blood vessels. They also transport glutamate into soma and simultaneously detoxicate glutamine by converting toxic OH_2_ into less harmful H_2_O_2_. These findings suggest that glial cells can exert protective effects and increase neuronal survival through their trophic, siphoning, and detoxicating actions.

Ischemic stroke has been associated with marked cell damage characterized by widespread activation of glial cells, or reactive gliosis. However, there is a debate as to whether such gliosis is a response to cell death or an early neuroprotective response. In experimental models of ischemia, some studies have reported that astrocytes are more resistant than neurons [73,74], while other investigations provide equally compelling evidence suggesting that astrocytes are of higher vulnerability than neurons [75,76]. Because of the presence of dense glial cell accumulations in the ischemic penumbra, their role in propagation or limitation of infarction size is widely argued [65]. Notwithstanding, the highly glial cell-populated ischemic penumbra has been suggested to be a conducive target site for cellular treatment intervention [77,78]. Transplantation of fetal [79,80] or cultured neurons [81], near or within the ischemic penumbra, has been found to induce behavioral recovery in ischemic animals. In the clinic, the ischemic penumbra is also targeted by anticoagulants, or thrombolytics, to dissolve blood clots [10]. Although drug therapy remains the preferred treatment for stroke patients, there has been no conclusive evidence of long-lasting motor and cognitive improvement with any of the current drugs [10]. Thus, stroke remains a leading cause of death in the world, and finding ways to rescue the central nervous system after ischemia, remains a major research endeavor.

Because of obvious alterations in glial cells after cerebral ischemia, we hypothesized and explored that if melatonin elicited therapeutic effects against cerebral ischemia, then it could also exert protective actions on glial cells and neurons [52]. Our *in vitro* and *in vivo* data demonstrated that the protection of glial cells afforded by melatonin led to functional recovery in stroke animals [52]. The reductions in glial cell loss and gliosis in melatonin-treated ischemic animals were paralleled by the observations of near normal motor functions in these animals. The ischemia-induced behavioral deficits seem to be mediated largely by a functional cortex in the melatonin-treated ischemic animals, which had minimal cortical infarction compared to saline-treated ischemic animals. Even though these animals also displayed a reduction in total striatal infarction, the lateral aspect of the striatum was still clearly damaged, suggesting that protection of the cortex may be sufficient for normalization of motor behaviors. The absence of behavioral protection by melatonin during the 1 h occlusion indicates that the drug (administered once before the arterial occlusion) did not block the functional deficits associated with the acute ischemic insult caused by interruption of cerebral blood flow. It appears that melatonin was protective against secondary cell death processes. The positive *in vivo* effects of melatonin were replicated *in vitro*, and demonstrated through continued survival of astrocytes treated with melatonin in following serum deprivation or toxin exposure (3-NP and Sodium Nitroprusside), which paralleled some *in vivo* cellular events observed in response to ischemia/reperfusion injury.

As noted above, a widely accepted mechanism for the protective action of melatonin involves a direct free radical scavenging effect on neurons. Melatonin is a highly potent free radical scavenger [82], and its administration to rats has been found to be effective against neurotoxicity [83–85]. Recent reports have demonstrated protective effects of melatonin against experimental ischemic damage [39,40,86–88] and a deficiency in melatonin has been suggested in stroke patients [89]. Melatonin is an effective free radical scavenger and indirect antioxidant [39,83–87,90–92]. Hydroxyl radicals (generated by hydrogen peroxide via the Fenton reaction) and peroxynitrite anions are scavenged by melatonin [91]. In addition, melatonin blocks singlet oxygen-induced toxicity [93]. Lipid peroxidation in the brain, produced by intoxication of free radical generating agents, is also reduced by melatonin [85,94]. These studies demonstrate that melatonin directly protects neural tissue from free radical toxicity.

The above studies, however, have not examined alterations in glial cells following melatonin treatment. Our findings advanced the concept that enhanced survival of glial cells after melatonin treatment may confer protection to injured neurons. The enveloping action of glial cells on neurons, might aid in the homeostasis of the neuronal cell membrane by siphoning excess potassium or by enhancing water handling capacity. In addition, glial cells may serve as cystine/glutamate antiporter systems that can prevent glutamate toxicity [57,58,95]. Finally, glial cells can secrete trophic factors, including GDNF, which has recently been shown to protect against experimental cerebral ischemia [96]. Thus, the combined buffering action, anti-glutamate toxicity transporter mechanism, and trophic factor-secreting potential of glial cells makes them efficacious neuroprotective agents, which could be recruited by melatonin to combat ischemia/reperfusion injury.

## 3. Melatonin and Stroke: Transplantation of Pineal Gland in Experimental Stroke Animals

Over the last two decades, cell replacement therapy has been proven effective in many animal models of neurological disorders, as well as in clinical settings [97–100]. In the laboratory, different types of cells, such as fetal striatal or cortical cells, genetically engineered cells, and stem cells, have been transplanted into experimental stroke animal models and shown beneficial effects [80,101,102]. The world’s first clinical trial of neural transplantation therapy for stroke was initiated in 1998 [103]. In this pioneering clinical study, human-derived cells (called NT2N cells), which exhibit neuronal features, were transplanted near the stroke area in hope that the cells would replace dead or dying host brain cells, and alternatively exert neurotrophic or anti-inflammatory effects. Encouraging clinical data [104,105] suggest that intracerebral transplantation therapy is feasible for stroke. Recent clinical trials have also explored intravenous transplantation of stem cells in acute stroke patients [106,107].

To this end, we examined whether pineal gland grafts promoted neuroprotective effects in rats exposed to acute stroke model [53]. Stroke rats that received rat-derived pineal gland allografts displayed significantly less motor asymmetry and reduced cerebral infarction than control stroke rats that did not receive the transplants. This observed neuroprotection was achieved when the host pineal gland was intact, in that pinealectomy blocked the protective effects of pineal gland grafts. Furthermore, such pineal gland graft-induced neuroprotection was accompanied by elevations in CSF melatonin. The observed neuroprotection produced by pineal gland grafts paralleled our earlier observation of beneficial effects following chronic, exogenous melatonin administration in the same stroke model [45,52]. In these studies, a similar amelioration of motor and histological deficits was observed in melatonin-treated stroke animals. Melatonin-induced neuroprotection also has been demonstrated in other models of stroke and CNS disorders [108–110].

The question arises then whether intracerebral grafting of pineal glands is as efficacious as exogenous melatonin treatment for stroke. Of note, the observed reduction of infarct size was apparent at days 2 and 3 poststroke, but not at day 1 [53]. This suggests that pineal gland grafts primarily targeted secondary cell death (*i.e*., apoptotic cell death) as opposed to the initial stroke insult (*i.e*., necrotic cell death). Because secondary cell death ensues after onset of stroke [9,10,111], chronic melatonin treatment is indicated for maintained therapeutic efficacy. Direct comparisons between exogenous melatonin and pineal gland grafts may reveal the better treatment option, however, the benefit–risk ratio needs to be considered. For example, pineal gland grafts involve an invasive surgical procedure, whereas exogenous melatonin does not expose the subject to such trauma. On the other hand, with inherent massive cell loss following stroke [108–110,112,113], exogenously rescuing or stimulating spared cells in the ischemic area may prove less effective when compared to cell replacement therapy through pineal gland grafts. Alternatively, each treatment may be catered to the disease stage, in that exogenous melatonin may be appropriate for early acute stroke treatment, while pineal gland grafts may target chronic stroke. Additionally, a combination of both treatment regimens may yield enhanced functional outcomes. It should be noted that our previous study [53] investigated the acute phase of post-transplantation and did not use immunosuppressive agents, but there may be a need for these agents when long-term graft survival is indicated for stable functional recovery with pineal grand transplantation. While the brain was classically thought to be “immune privileged,” graft rejection can still occur. Thus, due consideration is necessary when assessing the clinical utility of porcine pineal gland/cell line transplantation for stroke patients.

Examination of the mechanism of action underlying pineal gland grafts may require the need for co-administration of melatonin antagonist or free radicals during transplantation, or the use of non-melatonin secreting tissues as negative control grafts to reveal interactions between pineal gland grafts and melatonin. The possibility exists that pineal gland grafts also might have secreted growth factors to exert neuroprotection; this hypothesis can be similarly examined by coadministration of antibodies directed against growth factors during transplantation as we have done previously to elucidate a trophic factor-mediated mechanism in other graft sources (e.g., fetal kidneys, testis-derived Sertoli cells) [97,114–116]. Finally, future studies are warranted to extend the therapeutic efficacy of pineal gland grafts in a chronic stroke model and to characterize functional outcomes in the long term.

Finally, we have demonstrated pinealectomy blocked pineal gland graft-induced neuroprotection [53]. This observation suggests possible interactions between host and grafted glands, such as forming a neural network as has been demonstrated in many transplant studies [99,117–119], which may promote repair of the ischemic area. However, because the transplant site for the pineal glands was the striatum, which is distantly located from the host intact pineal gland, it is unlikely that neural connections formed between the two tissues. Moreover, the short 3 days of graft maturation would limit any axonal sprouting from either transplant or host pineal gland. The most plausible explanation for requiring an intact host pineal gland to promote neuroprotection in pineal gland grafts is the significantly elevated level of melatonin produced by both endogenous and exogenously transplanted pineal glands compared with those in pinealectomized transplanted or vehicle-infused animals. Accordingly, a high level of melatonin needs to be available in the brain to exert neuroprotection. Furthermore, if a sustained high level of melatonin is required to provide optimal neuroprotection, pineal gland grafts may provide better neuroprotection than exogenously delivered melatonin. A pineal gland graft can secrete a constant amount of melatonin in the brain [5,120], whereas the latter may allow only transient bursts of elevated melatonin levels in the brain upon administration. Intracerebral minipump infusion of melatonin may circumvent such brain delivery problems with constant drug dose. However, the obtrusive surgical procedure with this minipump regimen indicates that the equally invasive intracerebral pineal gland grafts may prove more advantageous in that microenvironmental cues (e.g., free radicals, inflammatory responses) are available to the grafts, which may help to modulate the appropriate level of melatonin secretion. Thus in the end, it may not be that high levels of melatonin exclusively are needed for optimal neuroprotection, but rather a “dynamic” level of melatonin over the stroke progression is most favorable. Indeed, aberrant accumulation of free radicals during stroke has been well documented in stroke animals and patients [12,13,121–123]. In addition, inflammation, which could act as an exacerbating or limiting factor for stroke [122,124,125], may influence graft survival. As we [4,52], and several others [6–8], have hypothesized, the neuroprotection of pineal glands is underlined by its free radical scavenging property. Furthermore, in any cell transplantation regimen, neuroprotection largely depends on the rescue of the host microenvironment [97,98,126]. With these critical factors in mind, fluctuations in levels of stroke-induced free radicals, and inflammatory elements in the host brain [127–129] may therefore serve as endogenous cues for dynamic secretion of melatonin by pineal gland grafts, achieving optimal neuroprotection.

Intracerebral transplantation of pineal glands, as well as pinealectomy, has been previously performed in animals to demonstrate the participation of this brain organ in modulating circadian rhythms [1–3,130,131]. The feasibility of pineal gland transplantation, together with the use of other novel cells for transplantation [79,115,116,126,132,133], partially provided the impetus of using pineal glands as graft source for stroke therapy. Neuroprotection by pineal gland grafts suggests that using porcine pineal glands, or establishing pineal gland cell lines, may be the next step towards clinical application of this transplantation therapy for stroke. Feasibility and efficacy of pineal glands as an alternative graft source for neural transplantation therapy, also provides direct evidence of central effects of free radical scavengers in the injured brain. Investigations into developing melatonin analogues may prove equally beneficial for stroke therapy.

## 4. Melatonin Action on Stem Cells: Involvement of Specificmelatonin Receptor

To our knowledge, we are one of the first to report the role of melatonin receptors in stem cell therapy [54], which we have discussed here. We also acknowledge that there are many other mechanisms with which stem cell contributes to the neuroprotection in stroke, including the secretion of trophic factors [134]. In our quest to find a crosstalk in neuroprotective pathways, in particular between melatonin and cell therapies, we recently examined melatonin receptor expression in stem cells. Amniotic epithelial cells (AEC) are pluripotent stem cells and can easily be obtained from placental tissue and amniotic fluid [135–138]. Research focus on the use of the cells has turned toward neural function as well as use of AECs to treat intracerebral hemorrhage and ischemia [137,138]. However, these studies have yet to demonstrate regulatory pathways underlying AEC differentiation, although a few studies have recently addressed this gap in our knowledge [139]. Moreover, there is paucity of information in providing evidence to support AEC therapeutic benefits in a particular neurological disease.

Numerous studies have documented melatonin-induced neuroprotection against ischemic and hemorrhagic stroke [52,140–145]. Postulated mechanisms of action of melatonin include preventing apoptosis [146–148] and reducing oxidative stress [149–153]. The notion that melatonin promotes neuroprotection via endogenous neurogenesis involving stem cells [147,154,155] is appealing to us because of our long-standing interest in cell therapy [53]. Stem cells express specific melatonin receptors 1 (MT1), and/or melatonin receptor 2 (MT2). These receptors are modulated by the melatonin ligand. However, investigation into the cross-talk between melatonin and stem cells is an under explored research area [156,157].

Accordingly, we investigated mechanisms underlying neural differentiation of AECs, and assessed the cells’ potential to afford neuroprotection in an experimental *in vitro* model of stroke [54]. As a result, we made five major observations. First, we demonstrated that AECs express MT1 receptors, but not MT2 suggesting that specific targeting of MT1 could alter the eventual fate of AEC. Similarly, a previous study [157] shows expression of MT1 in neural stem cells, implicating melatonin as a pleiotropic molecule in mammalian neurodevelopment. The second observation demonstrated that antagonizing of MT1, but not MT2, suppressed neuroprotective effect of AECs. The third major finding was that melatonin enhanced AEC proliferation and differentiation, specifically those AECs expressing MT1. Melatonin primes differentiation of neural stem cells [154] and of AECs expressing MT1, highlighting the importance of melatonin receptor-ligand mechanism in regulating neuronal function. While there are studies which reported therapeutic outcomes following exogenous melatonin treatment [143,144,158], our study provides a complementary approach of combining melatonin and AECs for achieving more effective neuroprotection [53,54]. Fourth, our study suggests applying AEC-melatonin combination treatment for diseases characterized by oxidative stress [47,144,147,150,159–162]. In addition to the effects of melatonin on cell proliferation and differentiation, it should also suppress neurodegeneration at different stages of cell death via anti-oxidative properties. Accordingly, benefits from the combined AEC-melatonin therapy seem to be better than AEC alone or melatonin monotherapy. Fifth, our study demonstrated involvement of neurotrophic factors in AEC-melatonin induced neuroprotection. VEGF was determined to be upregulated by melatonin treatment, providing further insights into the contribution of growth factors to the therapeutic outcome. Interaction between melatonin and VEGF has been previously demonstrated in the periphery [163] as well as another trophic factor BDNF in the cerebellar neurons [164]. We extend these past reports by showing elevated VEGF levels in AECs, and further clarification that BDNF appears to be closely associated with MT2 receptor expression [164], while VEGF correlates highly with MT1 receptor expression [54].

Our study directly implicated MT1 as a critical receptor for stem cell fate, whereas some studies have suggested the interaction between melatonin receptors and neurons [53,142,156,161,164–167]. Contribution of MT1 in stem cell function appears to be supported by the finding that reduced mammary tumor growth is associated with higher melatonin and MT1 receptors [165]. Our *in vitro* study presents many possibilities for further research, including assessments of neuroprotective effects of AECs and melatonin in *in vivo* brain disease models. In summary, stimulation of the melatonin receptor exerted a neuroprotective effect. Furthermore, AEC co-treatment with melatonin promoted a synergistic neuroprotective effect that was primarily mediated by stimulation of MT1. These results, taken together, advance the concept of melatonin receptor technology in stem cell therapy, and that stem cells can be switched on with melatonin or relatively switched off without melatonin to regulate its growth, differentiation, and secretion of growth factor. One can envision this melatonin receptor technology for efficient regulation of stem cell fate and function after transplantation in translational and/or clinical researches.

In addition to our observation of melatonin receptor participation in stem cell transplantation, there is considerable evidence highlighting roles for both MT1 and MT2 in neuroprotection outside of their potential effects in grafted stem cells [168,169]. This may be of particular interest given that there are already clinically available melatonin receptor agonists (e.g., Ramelteon) with better pharmacokinetic properties (*i.e*., plasma half-life, MT specificity/affinity) than melatonin itself [170,171].

## 5. Towards Clinical Applications of Melatonin-Based Therapeutics

The preceding sections discussed the potential of exogenous melatonin treatment, transplantation of melatonin-expressing pineal gland, and stimulating the melatonin receptor MT1 in stem cells, altogether advancing the efficacy of melatonin-based therapeutics for stroke. However, in order to proceed with clinical trials in any of these experimental strategies, the demonstration of safety of melatonin-based therapeutics is of high importance. Long-term monitoring of treated stroke animals for observation of any overt behavioral adverse effects, as well as examination of brain tissues and peripheral organs for any toxic effects, will be necessary to build the safety profile of the these treatments. We refer also to the STAIR [172] and STEPS [173,174] recommendations for enabling these therapeutics towards clinical entry. Among the translational guidelines outlined by these two stroke committees, the need to test the therapeutics in two models/species of stroke, the incorporation of co-morbidity factors (e.g., diabetes, hypertension, aging, *etc*.), multiple lab testing, and appropriate standard of care controls, will be critical to improving the successful entry of melatonin-based therapeutics in the clinic.

## 6. Conclusions

**Figure 1:**
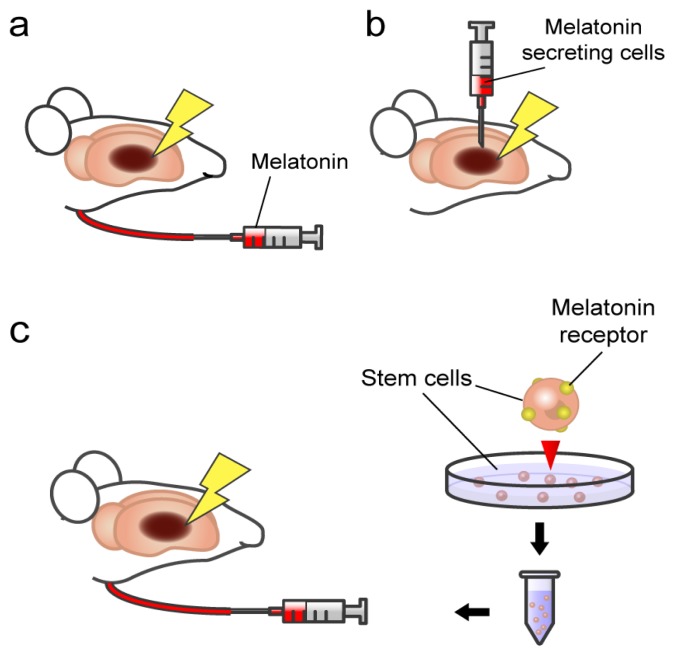
Schematic illustrations of melatonin therapies for stroke. (**a**) Exogenous melatonin administration; (**b**) Transplantation of pineal gland cells secreting melatonin; (**c**) Transplantation of stem cells expressing melatonin receptors. Combination of these therapies may prove advantageous in abrogating brain damage with stroke.

We highlight here that with the different pathways of cell death associated with stroke, that may involve a breakdown in the crosstalk between glia and neurons, and the progressive nature of stroke-induced secondary cell death including neurodegeneration, the optimal therapeutic regimen may require a combination treatment rather than a stand-alone treatment. Accordingly, the combination of exogenous melatonin treatment, transplantation of pineal gland, and stimulation of MT1 receptor in stem cells, together with other available stroke therapeutics (tPA), may prove advantageous in abrogating brain damage and behavioral deficits associated with stroke (Figure 1).

## Figures and Tables

**Table 1:** Recent publications demonstrating the effects of antioxidants on experimental models of stroke.

Author	Antioxidant	Model	Main finding
Qi *et al*. (2010) [14]	Leonurine	Rat/MCAo	Histological/functional improvement, inhibit ROS production
Loh *et al*. (2010) [15]	Leonurine	Rat/MCAo	Histological/functional improvement, inhibit ROS production
Thaakur *et al*. (2010) [16]	Spirulina	Rat/MCAo	Histological/functional improvement
Khan *et al*. (2010) [17]	Sesamin	Rat/MCAo	Functional improvement, reducing thiobarbituric acid reactive species and protein carbonyl
He *et al*. (2010) [18]	Parthenocissin A	Rat/MCAo	Histological/functional improvement, suppressing lipid peroxidation and restoring superoxide dismutase, inhibiting NO and NOS elevation
Simao *et al*. (2011) [19]	Resveratrol	Rat/Global cerebral ischemia	Reducing neuronal death and generation of ROS, lipid peroxidation and NO content
Zhang *et al*. (2011) [20]	Gypenosides	Rat/Chronic cerebral hypoperfusion	Improving cognitive function
Gaur *et al*. (2011) [21]	Hesperidin	Rat/Common carotid artery occlusion	Functional improvement, reducing oxidative damage
Ahmad *et al*. (2011) [22]	Quercetin dihydrate	Rat/MCAo	Histological/functional improvement
Tai *et al*. (2011) [23]	Melatonin	Primary neuron/OGD	Synergistic antioxidant and radical-scavenging actions with estradiol
Jung *et al*. (2011) [24]	Joongpoongtang 05	Rat/MCAo	Histological improvement, a decrease in oxidants
Suzuki *et al*. (2011) [25]	*Phellinus linteus* broth culture	Rat/MCAo	Histological improvement
Silachev *et al*. (2012) [26]	SkQR1	Rat/MCAo	Histological/functional improvement
Li *et al*. (2012) [27]	Galangin	Rat/MCAo	Histological/functional improvement, protective effect on the mitochondria
Park *et al*. (2012) [28]	Coenzyme Q10	NSC/hypoxia	Cell protection
Gundimeda *et al*. (2012) [29]	Green tea polyphenols	PC12 cell/OGD	Cell protection
Huang *et al*. (2012) [30]	MnTm4PyP	Mouse/MCAo, Cortical neurons/H_2_O_2_ injury	Histological/functional improvement and increased cell viabillity
Chen *et al*. (2012) [31]	Octreotide	Rat/MCAo	Histological/functional improvement, upregulation of transcription factor Nrf2, HO-1 and downregulation of NF-κB expression
Qian *et al*. (2012) [32]	Genistein	Mouse/MCAo	Histological/functional improvement, inhibits ROS production
Sakata *et al*. (2012) [33]	Minocycline	Rat/MCAo with pre-conditioned NSC transplantation, pre-conditined NSC/OGD	Histological/functional improvement, releasing paracrine factors from pre-conditioned NSCs
Connell and Saleh (2012) [34]	Apocynin, lipoic acid	Rat/MCAo	Histological improvement
Bae *et al*. (2013) [35]	Carnosine	Rat/MCAo	Histological/functional improvement

MCAo: Middle cerebral artery occlusion, OGD: Oxygen glucose deprivation, NSC: Neural stem cell, ROS: Reactive oxygen species.

**Table 2:** Clinical trials of antioxidants for treatment of stroke.

Sponsor	Condition	Drug	Start year	Completion year	Outcome (If available)
AstraZeneca	Cerebral Stroke	NXY-059	2003	2005	Ineffective for the treatment of acute ischemic stroke within 6 h after the onset of symptoms [36].
AstraZeneca	Cerebral Stroke	NXY-059	2003	2006	
Mitsubishi Tanabe Pharma Corporation	Cerebral infarction	Edaravone, Sodium Ozagrel	2004	2006	Edaravone was at least as effective as ozagrel for the treatment of acute noncardioembolic ischemic stroke [37].
Combination Therapy for Acute Ischemic Stroke Study Group	Stroke	Edaravone combined with argatroban	2004	2008	No favorable effects of edaravone when added to the baseline treatment with argatroban [38].
Mitsubishi Tanabe Pharma Corporation	Acute ischemic stroke	MCI-186	2009	2010	Not available
Otsuka Beijing Research Institute	Cerebral infarction	Cilostazol, Probucol	2009	2010	Not available
University of Science Malaysia	Cerebrovascular disorders	Palm vitamin E (tocotrienol)	2008	2012	Not available
University of Nottingham	Stroke	Transdermal glyceryl trinitrate patch (combined with prestroke antihypertensives)	2001	Ongoing	-
Asan Medical Center	Brain ischemia, Intracranial hemorrhages	Cilostazol, Probucol, Aspirin	2009	Ongoing	-
Brigham and Women’s Hospital	Stroke	Quercetin	2009	Ongoing	-
Takeda Global Research & Development Center, Inc.	Cardiovascular disease	Febuxostat, Allopurinol	2010	Ongoing	-
Angel Chamorro	Acute ischemic stroke	Uric acid	2011	Ongoing	-
Chandan K Sen	Transient ischemic stroke	Vitamin E tocotrienol (TCT) pills, Low dose Aspirin	2012	Ongoing	-
Nycomed: A Takeda company	Post-stroke cognitive impairment	Actovegin	2012	Ongoing	-
Zhejiang Hospital	Stroke	Aspirin, Warfarin, Atrvastatin, Edaravone (combined with autologous hematopoiesis stem cell transplantation)	2012	Ongoing	-
